# What do Cochrane systematic reviews say about cardiac arrest management?

**DOI:** 10.1590/1516-3180.2018.0083230318

**Published:** 2018-01-09

**Authors:** Rafael Leite Pacheco, Juliana Trevizo, Caio Augusto de Souza, Gabriel Alves, Bruno Sakaya, Luciana Thiago, Aécio Flávio Teixeira de Góis, Rachel Riera

**Affiliations:** I MD. Volunteer Researcher at Cochrane Brazil, São Paulo (SP), Brazil.; II Undergraduate Medical Student, Escola Paulista de Medicina - Universidade Federal de São Paulo (EPM-Unifesp), São Paulo (SP), Brazil.; III Undergraduate Medical Student, Escola Paulista de Medicina - Universidade Federal de São Paulo (EPM-Unifesp), São Paulo (SP), Brazil.; IV Undergraduate Medical Student, Escola Paulista de Medicina - Universidade Federal de São Paulo (EPM-Unifesp), São Paulo (SP), Brazil.; V Undergraduate Medical Student, Escola Paulista de Medicina - Universidade Federal de São Paulo (EPM-Unifesp), São Paulo (SP), Brazil.; VI MD, MSc, PhD. Cardiologist and Medical Preceptor, Discipline of Evidence-Based Medicine, Escola Paulista de Medicina - Universidade Federal de São Paulo (EPM-Unifesp), São Paulo, Brazil.; VII MD, MSc, PhD. Cardiologist and Adjunct Professor, Discipline of Evidence-Based Medicine, Escola Paulista de Medicina - Universidade Federal de São Paulo (EPM-Unifesp), São Paulo, Brazil.; VIII MD, MSc, PhD. Rheumatologist and Adjunct Professor, Discipline of Evidence-Based Medicine, Escola Paulista de Medicina - Universidade Federal de São Paulo (EPM-Unifesp); Volunteer Researcher, Cochrane Brazil, São Paulo (SP), Brazil.

**Keywords:** Review [publication type], Heart arrest, Evidence-based medicine, Evidence-based practice

## Abstract

**CONTEXT AND OBJECTIVE:**

Cardiac arrest is associated with high morbidity and mortality and imposes a significant burden on the healthcare system. Management of cardiac arrest patients is complex and involves approaches with multiple interventions. Here, we aimed to summarize the available evidence regarding the interventions used in cardiac arrest cases.

**DESIGN AND SETTING:**

Review of systematic reviews (SRs), conducted in the Discipline of Evidence-Based Medicine, Escola Paulista de Medicina, Universidade Federal de São Paulo.

**METHODS:**

A systematic search was conducted to identify all Cochrane SRs that fulfilled the inclusion criteria. Titles and abstracts were screened by two authors.

**RESULTS:**

We included nine Cochrane SRs assessing compression techniques or devices (three SRs), defibrillation (two SRs) and other interventions (two SRs on hypothermia interventions, one on airway management and one on pharmacological intervention). The reviews included found qualities of evidence ranging from unknown to high, regarding the benefits of these interventions.

**CONCLUSION:**

This review included nine Cochrane systematic reviews that provided a diverse range of qualities of evidence (unknown to high) regarding interventions that are used in management of cardiac arrest. High-quality evidence was found by two systematic reviews as follows: (a) increased survival until hospital discharge with continuous compression, compared with interrupted chest compression, both administered by an untrained person and (b) no difference regarding the return of spontaneous circulation, comparing aminophylline and placebo, for bradyasystolic patients under cardiac arrest. Further studies are needed in order to reach solid conclusions.

## INTRODUCTION

Sudden cardiac arrest is an important cause of death and is responsible for 15% of total mortality in the United States.^1^ Its occurrence is associated with a poor prognosis, despite the numerous interventions that are available for treating this condition.^2^

Occurrences of sudden cardiac arrest are usually associated with an underlying structural heart disease, and coronary heart disease is the most frequent form (two thirds of the cases). Other heart diseases such as myocarditis and hypertrophic cardiomyopathies are also common causes. When there is no structural disease, most cases occur due to arrhythmia, for which there are very many etiologies.^1^^,^^3^^,^^4^

Several criteria have been used to define cardiac arrest in the medical literature. In 2006, the American College of Cardiology/American Heart Association/Heart Rhythm Society (ACC/AHA/HRS) established the standard definition for cardiac arrest as “sudden cessation of cardiac activity so that the victim becomes unresponsive, with no normal breathing and no signs of circulation. If corrective measures are not taken rapidly, this condition progresses to sudden death”. In current clinical practice, cardiac arrest is reversed mainly by cardiopulmonary resuscitation and/or cardioversion or defibrillation, or cardiac pacing.^5^

Despite the importance of cardiac arrest, there is uncertainty regarding the use of most interventions that have been recommended for its management. Several guidelines for its treatment are available, but an analysis of the best evidence is always useful, to guide further studies and to update the recommendations with the best unbiased evidence. Hence, synthesis studies such as the present review are imperative for enabling critical analysis and for summarizing the results from primary research on cardiac arrest patients.

The aim of the present review was to identify and summarize the evidence from Cochrane systematic reviews (SRs) relating to interventions for managing cardiac arrest in any setting.

## METHODS

### Design and setting

This was a review of Cochrane systematic reviews (SRs), conducted within the Discipline of Evidence-Based Medicine of Escola Paulista de Medicina, Universidade Federal de São Paulo (EPM-Unifesp). This article was specifically developed for the section Cochrane Highlights, which is an initiative for disseminating Cochrane reviews. This initiative results from a formal partnership between the São Paulo Medical Journal and Cochrane, and it is supported by Cochrane Brazil.

### Inclusion criteria

#### Types of study

We included SRs published in the Cochrane Database of Systematic Reviews (CDSR). Protocols for SRs and withdrawn or previous versions of single SRs were not included. No limit for publication date was applied.

#### Types of participants

The participants comprised patients who had been diagnosed as presenting cardiac arrest, regardless of the setting (pre-hospital or in-hospital) or their age or sex.

#### Types of intervention

We considered SRs assessing any intervention (either pharmacological or non-pharmacological), whether applied separately or combined with others.

#### Types of outcomes 

We considered any clinical or laboratory outcome, as evaluated by the authors of the SRs included.

### Search for reviews

We conducted a sensitive search in the Cochrane Database of Systematic Reviews (CDSR) (via Wiley) on February 24, 2018, using the MeSH term “Heart Arrest” and all related variants, in titles, abstracts and keywords. The detailed search strategy is presented in Table 1.

**Table 1:** Search strategy

#1 MeSH descriptor: [Heart Arrest] explode all trees (in titles, abstracts and keywords)
#2 (Arrest, Heart) OR (Cardiac Arrest) OR (Arrest, Cardiac) OR (Cardiopulmonary Arrest) OR (Arrest, Cardiopulmonary) OR (Heart Arrest, Induced ) OR (Induced Heart Arrest) OR (Cardiac Arrest, Induced) OR (Induced Cardiac Arrest) OR (Out-of-Hospital Cardiac Arrest) OR (Cardiac Arrest, Out-of-Hospital) OR (Cardiac Arrests, Out-of-Hospital) OR (Out of Hospital Cardiac Arrest) OR (Out-of-Hospital Cardiac Arrests) OR (Out-of-Hospital Heart Arrest) OR (Heart Arrest, Out-of-Hospital) OR (Heart Arrests, Out-of-Hospital;) OR (Out of Hospital Heart Arrest) OR (Out-of-Hospital Heart Arrests) OR (Heart Massages) OR (Massage, Heart) OR (Massages, Heart) OR (Sinus Arrest, Cardiac) OR (Cardiac Sinus Arrests) OR (Sinus Arrests, Cardiac) OR (Cardiac Sinus Arrest)
#3 #1 OR #2
#4 #3 Filter: in Cochrane Reviews

### Selection of reviews

The titles and abstracts were screened by two authors (RLP and RR) independently. Any disagreements were resolved by reaching a consensus. The SRs that met the inclusion criteria were selected and summarized by five authors (RLP, JT, CAS, BS, GA).

### Presentation of results

The results from the search and the SRs included were presented through a narrative approach (qualitative synthesis).

## RESULTS

### Search results

Our search strategy retrieved 543 references and, after screening the titles and abstracts, nine systematic reviews (SRs) were found to fulfill our inclusion criteria and were considered for qualitative synthesis.^6^^,^^7^^,^^8^^,^^9^^,^^10^^,^^11^^,^^12^^,^^13^^,^^14^

### Reviews included

We present a short individual summary of each SR included. Details about the characteristics of interventions, comparisons, outcomes and quality of evidence are presented in Table 2.

**Table 2:** Characteristics of interventions, comparisons, outcomes and quality of evidence

Compression techniques or devices				
Intervention	Comparators	Population	Main findings	GRADE^17^
Active chest compression-decompression (ACDR)^6^	Standard cardiopulmonary resuscitation (SCR)	Out-of-hospital or in-hospital cardiac arrest patients	Similar results were found for out-of-hospital and in-hospital cardiac arrest: there were no differences between the groups regarding mortality up to hospital discharge, neurological impairment or complications (such as fractures and pneumothorax and hemothorax).	Not assessed
Mechanical chest compression^10^	Manual (standard) chest compression	Cardiac arrest patients	One RCT found improved neurological function and survival until hospital discharge, favoring mechanical chest compression. This result was inconsistent with others included in the RCT but no quantitative synthesis was performed because of heterogeneity of the data.	Very low to moderate
Continuous chest compression^12^	Interrupted chest compression	Non-asphyxial out-of-hospital cardiac arrest	When performed by an untrained person, continuous chest compression achieved higher rates of survival until hospital discharge but no difference in neurological outcomes. When performed by a trained person, there was no difference between the groups regarding survival or neurological outcomes.	Moderate to high
Defibrillation				
Intervention	Comparators	Population	Main findings	GRADE^17^
Biphasic transthoracic defibrillation^8^	Monophasic transthoracic defibrillation	Out-of-hospital cardiac arrest	No difference between the groups regarding survival until hospital discharge. No difference regarding failure to defibrillate and return of spontaneous circulation.	Not assessed
Delayed defibrillation^13^	Immediate defibrillation	Out-of-hospital cardiac arrest	No difference between the groups was found regarding survival until hospital discharge, good neurological outcome and return of spontaneous circulation.	Low
Other interventions				
Intervention	Comparators	Population	Main findings	GRADE^17^
Aminophylline^9^	No intervention	Bradyasystolic cardiac arrest	No difference between the groups regarding survival until hospital discharge and return of spontaneous circulation.	Low to high
Pre-hospital cooling^7^	In-hospital cooling	Cardiac arrest patients	There was a lack of data for quantitative synthesis, but the individual RCTs included did not find differences between the groups.	Very low
Hypothermia^11^	No intervention	Cardiac arrest patients	Conventional cooling was more likely to achieve a positive neurological outcome, increased survival and higher rates of adverse events (pneumonia and hypokalemia).	Low to moderate
Emergency intubation^14^	Other airway management techniques (bag-valve-mask ventilation, esophageal gastric tube or combi-tube)	Acutely ill and injured patients	For the comparison ETI versus bag-valve-mask ventilation and subsequently ETI, there was no difference between the groups regarding survival and good neurological outcome at hospital discharge. For the comparisons ETI versus esophageal gastric tube and ETI versus combi-tube, there was no difference in survival between the groups at hospital discharge.	Not assessed

RCTs = randomized clinical trials; ETI = endotracheal intubation. *GRADE (Grading of Recommendations Assessment, Development and Evaluation) has the aim of assessing the quality of the evidence. From this, the results are classified as having high quality of evidence (high confidence that the estimated effect is close to the true effect); moderate quality of evidence (likely that the estimated effect is close to the real effect but there is a possibility that it is not); low quality of evidence (limited confidence in the effect estimate) or very low quality of evidence (the true effect is likely to be substantially different from the estimate effect).

### Compression techniques or devices

#### Active chest compression-decompression with a hand-held suction device

This review^6^ had the aim of evaluating the use of active compression-decompression (ACD) for cardiopulmonary resuscitation (CPR), consisting of application of a hand-held suction device to the sternum. Ten randomized clinical trials (RCTs) were included, assessing either out-of-hospital settings (eight RCTs; 4,162 participants) or in-hospital settings (two RCTs; 826 participants).

Regarding out-of-hospital settings, no differences were found between active compression-decompression for cardiopulmonary resuscitation with a hand-held suction device and standard manual cardiopulmonary resuscitation (STR) regarding mortality either immediately (relative risk [RR] 0.98; 95% confidence interval [95% CI] 0.94 to 1.03; ten RCTs; 4,162 participants) or until hospital discharge (RR 0.99; 95% CI 0.98 to 1.01; nine RCTs; 3,412 participants). Despite the lack of long-term evaluation, there was no significant difference between the groups regarding severe neurological impairment (RR 3.11; CI 95% 0.98 to 9.83; four RCTs; 107 participants) or moderate neurological impairment (RR 0.98; CI 95% 0.34 to 2.79; four RCTs; 107 participants). Combined analysis (any neurological impairment) also found no difference (RR 1.71; CI 95% 0.90 to 3.25; five RCTs; 144 participants). There was no difference in the frequencies of complications (such as fractures and pneumothorax or hemothorax) between the groups (RR 1.09; 95% CI 0.86 to 1.38; seven RCTs; 3,032 participants).

The in-hospital analysis found similar results, although these results were only supported by two RCTs. No difference in immediate mortality (RR 0.98; 95% CI 0.88 to 1.08; two RCTs; 826 participants) or at-discharge mortality (RR 1.00; 95% CI 0.95 to 1.05; two RCTs; 826 participants) was found. The neurological impairment analysis found that there was no difference between the groups regarding moderate impairment (RR 0.5; 95% CI 0.10 to 2.59; two RCTs; 93 participants), severe impairment (RR 1.95; 95% CI 0.59 to 6.50; two RCTs; 93 participants) or impairment of any severity (RR 1.19; 95% CI 0.50 to 2.86; two RCTS; 93 participants). Nor was there any difference in the complications found (RR 0.97; 95% CI 0.49 to 1.93; one RCT; 773 participants).

The authors concluded that the use of ACD with a hand-held suction device for CPR was not associated with any benefit in relation to cardiopulmonary resuscitation.

For further details, refer to the original abstract, available at: http://onlinelibrary.wiley.com/doi/10.1002/14651858.CD002751.pub3/full.

#### Mechanical versus manual chest compression for cardiac arrest

This review^10^ assessed the efficacy and safety of mechanical chest compression in comparison with manual chest compression in cardiopulmonary resuscitation. Six RCTs were included (n = 1,166), although only one study reported the main clinical outcome of survival until hospital discharge with “good neurological function” (defined as cerebral performance category scores^15^ of 1 or 2). The group that underwent mechanical chest compression had shorter survival than the group with manual chest compression (RR 0.41; 95% CI 0.21 to 0.79; one RCT; 767 participants).

Three RCTs assessed survival until hospital discharge. Because of the clinical and methodological diversity between them, no pooled analysis was performed and the data were reported only narratively. One RCT reported a higher frequency of survival favoring the mechanical compression group (OR 2.81; 95% CI 1.26 to 6.24; one RCT; 152 participants) while the other two found that there was no difference between the groups: (OR 0.76; 95% CI 0.44 to 1.41; one RCT; 767 participants) and (OR 0.81; 95% CI 0.26 to 2.53; one RCT; 147 participants).

The authors concluded that there was insufficient evidence to reach any solid conclusion between the interventions evaluated. Further studies of good methodological quality with well-reported results would be needed to reduce the uncertainties.

For further details, refer to the original abstract, available at: http://onlinelibrary.wiley.com/doi/10.1002/14651858.CD007260.pub3/full.

#### Continuous versus interrupted chest compression for cardiopulmonary resuscitation of non-asphyxial out-of-hospital cardiac arrest

This review^12^ compared continuous versus interrupted chest compression for cardiopulmonary resuscitation among patients with non-asphyxial out-of-hospital cardiac arrest. Four RCTs were included (n = 26,742). The authors decided to divide the analysis into two groups: CPR performed by an untrained person or by a trained professional.

The analysis on CPR performed by an untrained person showed the following:

- Increased survival until the outcome of hospital discharge favoring the continuous chest compression group (RR 1.21; 95% CI 1.01 to 1.46; three RCTs; 3,031 participants).
- No difference between groups regarding neurological outcomes at hospital discharge (RR 1.25; 95% CI 0.94 to 1.66; one RCT; 1,286 participants).

The analysis on CPR performed by a trained professional was reported in one cluster RCT and showed the following:

- No statistical difference in the risk of survival between the group that received continuous chest compression (9.0%) and the group that received interrupted chest compression group (9.7%) (adjusted risk difference, ARD -0.7%; 95% CI -1.5% to 0.1%; one RCT; 23,711 participants).

The authors concluded that there was moderate to high quality of evidence supporting the use of continuous compression when CPR was performed by an untrained person. One large RCT showed that there was no statistically significant difference between the interventions when the CPR was performed by a trained professional.

For further details, refer to the original abstract, available at: http://onlinelibrary.wiley.com/doi/10.1002/14651858.CD010134.pub2/full.

### Defibrillation

#### Biphasic versus monophasic waveforms for transthoracic defibrillation in out-of-hospital cardiac arrest

This review^8^ aimed to compare the use of biphasic and monophasic defibrillators in situations of out-of-hospital cardiac arrest. It included 4 RCTs (n = 552). The main outcome was failure to achieve return of spontaneous circulation (ROSC), and no difference was found between the groups (RR 0.86; 95% CI 0.62 to 1.20; four RCTs; 552 participants). Failure to defibrillate was also assessed and there was no difference in relation to the first shock (RR 0.84; 95% CI 0.70 to 1.01; three RCTs; 450 participants), in relation to up to three shocks (RR 0.81; 95% CI 0.61 to 1.09; two RCTs; 317 participants) or in relation to failure to achieve ROSC after the first shock (RR 0.92; 95% CI 0.81 to 1.04; two RCTs; 285 participants). There was no difference in survival until hospital admission (RR 1.05; 95% CI 0.90 to 1.23; three RCTs; 383 participants) or in survival until hospital discharge (RR 1.05; 95% CI 0.78 to 1.42; four RCTs; 550 participants).

The authors concluded from the results from this review that there was a lack of precision in evaluations on biphasic and monophasic waveforms. The data showed that there was no benefit from using a biphasic defibrillator, although further studies would be warranted to increase the confidence in these results. For further details, refer to the original abstract, available at: http://onlinelibrary.wiley.com/doi/10.1002/14651858.CD006762.pub2/full.

#### Cardiopulmonary resuscitation (CPR) plus delayed defibrillation versus immediate defibrillation for out-of-hospital cardiac arrest

This review^13^ compared CPR plus delayed defibrillation versus immediate defibrillation among patients who suffered out-of-hospital cardiac arrest. Four RCTs were included (n = 3,090). There was no difference between the groups regarding survival until hospital discharge (RR 1.09; 95% CI 0.54 to 2.2; three RCTs; 658 participants), good neurological recovery at hospital discharge (RR 1.12; 95% CI 0.65 to 1.93; three RCTs; 2834 participants), return to spontaneous circulation (RR 0.94; 95% CI 0.77 to 1.15; three RCTs; 658 participants) or survival at one year afterwards (RR 0.77; 95% CI 0.24 to 2.49; two RCTs; 456 participants).

The authors’ conclusion was that the overall quality of evidence was low (mainly due to the risk of bias among the studies included and the imprecision of the results). There was no difference between the two interventions, and further studies would be needed to reduce the uncertainties of this analysis.

For further details, refer to the original abstract, available at: http://onlinelibrary.wiley.com/doi/10.1002/14651858.CD009803.pub2/full.

### Other interventions

#### Aminophylline for bradyasystolic cardiac arrest among adults

This review^9^ aimed to determine the effects (harm and benefits) of aminophylline administered to patients who suffered bradyasystolic cardiac arrest. Five RCTs (n = 1,186) were included. All of them were performed in pre-hospital settings.

There was no difference between aminophylline and placebo administration regarding the following outcomes:

- Survival until hospital discharge (odds ratio, OR 0.58; 95% CI 0.12 to 2.74; five RCTs; 1,254 participants).
- Return of spontaneous circulation (OR 1.15; 95% CI 0.89 to 1.49; five RCTs; 1,254 participants).
- Survival until admission (OR 0.92; 95% CI 0.61 to 1.37; five RCTs; 1,254 participants).

There were insufficient data to evaluate neurological outcomes and adverse events. The authors concluded that prehospital administration of aminophylline was not associated with any improvement in clinical outcomes. For further details, read the original abstract, available at: http://onlinelibrary.wiley.com/doi/10.1002/14651858.CD006781.pub3/full.

#### Pre-hospital versus in-hospital initiation of cooling for survival and neuroprotection after out-of-hospital cardiac arrest

This review^7^ evaluated the initiation setting (pre-hospital versus in-hospital) of cooling applied to cardiac arrest patients. Seven RCTs were included (n = 2,369). The authors’ aim was to assess the major clinical outcome of survival (short and long-term), along with neurological outcomes and safety outcomes (serious adverse events). Despite the considerable number of RCTs and participants, the authors did not perform any pooled analysis (quantitative synthesis) because of the existence of methodological heterogeneity. They stated that none of the RCTs found any statistical differences between the two intervention groups, but this may have been influenced by lack of statistical power and low event rates in single studies.

Further studies with good methodological quality and pre-planned outcomes need to be conducted to reduce the uncertainty regarding where to initiate cooling among patients who have suffered cardiac arrest. Another key point is that this review performed a head-to-head analysis. The use of hypothermia compared with inactive control was studied in another Cochrane systematic review, discussed below.^11^

For further details, and to access the full report on all the RCTs included, refer to the original abstract, available at: http://onlinelibrary.wiley.com/doi/10.1002/14651858.CD010570.pub2/full.

#### Hypothermia for neuroprotection among adults after cardiopulmonary resuscitation

The purpose of this review^11^ was to investigate the effects (efficacy and safety) of therapeutic hypothermia after cardiac arrest. Six RCTs (n = 1,412) were included. The main results were the following:

- Conventional cooling was more likely to achieve a positive neurological outcome (RR 1.94; 95% CI 1.18 to 3.21; four RCTS; 437 participants) than was no cooling.
- Conventional cooling also increased the survival (RR 1.32; 95% CI 1.10 to 1.65; three RCTs; 383 participants).
- The incidence of the adverse effect of pneumonia was higher in the intervention group (RR 1.15; 95% CI 1.02 to 1.30; two RCTs; 1,205 participants). There was also higher incidence of hypokalemia (RR 1.15; 95% CI 1.03 to 1.84; two RCTs; 975 participants).

The overall quality of the evidence was considered low to moderate. The authors concluded that hypothermia was beneficial for patients who suffered out-of-hospital cardiac arrest, but they emphasized that this intervention would need to be studied in other settings.

For further details, refer to the original abstract, available at: http://onlinelibrary.wiley.com/doi/10.1002/14651858.CD004128.pub4/epdf.

#### Emergency intubation for acutely ill and injured patients

This review^14^ aimed to assess endotracheal intubation (ETI) among acutely ill and injured patients (children and adults) who were unable to maintain adequate airways. The intention was to compare ETI with other airway management techniques. Three RCTs (n = 1,177) were included in a qualitative synthesis.

One RCT included 830 children (71% with non-traumatic cardiac arrest) and compared out-of-hospital ETI with bag-valve-mask ventilation and subsequent emergency department ETI. There was no difference regarding survival until hospital discharge (OR 0.82; 95% CI 0.61 to 1.11; one RCT; 830 participants) or good neurological outcome (OR 0.87; 95% CI 0.62 to 1.22; one RCT; 830 participants). 

Another RCT evaluated ETI versus use of an esophageal gastric tube and included 175 patients who suffered out-of-hospital non-traumatic cardiac arrest. There was also no difference between the groups regarding survival until discharge (RR 0.86; 95% CI 0.39 to 1.90; one RCT; 175 participants).

The last RCT included 172 patients and compared ETI with use of a combi-tube among adults who suffered out-of-hospital non-traumatic cardiac arrest. There was also no difference between the groups regarding survival until hospital discharge (RR 0.43; 95% CI 0.09 to 1.99; one RCT; 172 participants).

The authors concluded that the efficacy of emergency intubation had not been rigorously studied, despite its widespread use in current practice. Further studies would be needed to evaluate this intervention among cardiac arrest patients.

For further details, refer to the original abstract, available at: http://onlinelibrary.wiley.com/doi/10.1002/14651858.CD001429.pub2/full.

## DISCUSSION

This review included nine Cochrane systematic reviews that assessed interventions among patients who suffered cardiac arrest. The interventions included related to the use of compression techniques or devices (three SRs), defibrillation (two SRs) and other interventions (two SRs regarding hypothermia interventions, one regarding airway management and one regarding pharmacological intervention).

Although cardiac arrest is a very common cause of death, this condition has not been greatly studied through RCTs, i.e. using the gold-standard primary research design for evaluating the efficacy and safety of interventions. This may have happened partially because it is more difficult and very costly to perform RCTs under emergency conditions, and even more so during management of cardiac arrest. These interventions are commonly delivered by more than one person, which requires more training and elevates the clinical diversity between studies. Even the concept of “controlled” is challenged under these conditions, since most of the interventions are implemented in out-of-hospital settings, sometimes by an untrained person. This difficulty may be partly resolved by conducting clinical trials using nested designs, such as clustered designs, rather than using the widely used parallel design. However, this may lead to higher risk of bias and should be considered in planning further studies.

Regarding clinical implications, high-quality evidence was found in two systematic reviews as follows: (a) survival until hospital discharge is increased with continuous compression, when compared to interrupted chest compression, both administered by an untrained person and (b) there was no difference regarding the return of spontaneous circulation of bradyasystolic patients under cardiac arrest, comparing aminophylline and placebo. For all other comparisons and related outcomes, only very low to moderate evidence quality was found. Thus, clinical practice may be guided from the results presented in Table 2 and from those obtained through other study designs (especially well-performed comparative observational studies), but most of these findings may be subject to change in the light of data from future studies.

Regarding the implications for further research, it is highly necessary to ensure that any future RCT on interventions relating to cardiac arrest should be planned. Such studies should only assess clinically relevant outcomes. The reporting of such studies needs to rigorously follow the guidelines of the CONSORT^18^ statement, in order to enhance transparency and reproducibility.

## CONCLUSION

Most of the nine Cochrane systematic reviews assessing CPR found no evidence or only provided limited evidence to allow any practical recommendation. High-quality evidence was found by two systematic reviews as follows: (a) survival until hospital discharge was increased with continuous compression, when compared to interrupted chest compression, both administered by an untrained person; and (b) there was no difference regarding the return of spontaneous circulation of bradyasystolic patients under cardiac arrest, comparing aminophylline and placebo. Further studies are needed in order to reach solid conclusions.
